# Quantitative Peptidomics of *Purkinje Cell Degeneration* Mice

**DOI:** 10.1371/journal.pone.0060981

**Published:** 2013-04-08

**Authors:** Iryna Berezniuk, Juan J. Sironi, Jonathan Wardman, Raymond C. Pasek, Nicolas F. Berbari, Bradley K. Yoder, Lloyd D. Fricker

**Affiliations:** 1 Department of Neuroscience, Albert Einstein College of Medicine, Bronx, New York, United States of America; 2 Department of Molecular Pharmacology, Albert Einstein College of Medicine, Bronx, New York, United States of America; 3 Department of Cell, Development, and Integrative Biology, University of Alabama at Birmingham Medical School, Birmingham, Alabama, United States of America; University of Rouen, France, France

## Abstract

Cytosolic carboxypeptidase 1 (CCP1) is a metallopeptidase that removes C-terminal and side-chain glutamates from tubulin. The *Purkinje cell degeneration* (*pcd*) mouse lacks CCP1 due to a mutation. Previously, elevated levels of peptides derived from cytosolic and mitochondrial proteins were found in adult *pcd* mouse brain, raising the possibility that CCP1 functions in the degradation of intracellular peptides. To test this hypothesis, we used a quantitative peptidomics technique to compare peptide levels in wild-type and *pcd* mice, examining adult heart, spleen, and brain, and presymptomatic 3 week-old amygdala and cerebellum. Contrary to adult mouse brain, young *pcd* brain and adult heart and spleen did not show a large increase in levels of intracellular peptides. Unexpectedly, levels of peptides derived from secretory pathway proteins were altered in adult *pcd* mouse brain. The pattern of changes for the intracellular and secretory pathway peptides in *pcd* mice was generally similar to the pattern observed in mice lacking primary cilia. Collectively, these results suggest that intracellular peptide accumulation in adult *pcd* mouse brain is a secondary effect and is not due to a role of CCP1 in peptide turnover.

## Introduction

In the 1970s, a spontaneous mutant mouse was discovered and named *Purkinje cell degeneration (pcd)* due to the loss of cerebellar Purkinje cells starting around 3 weeks after birth [1]. A small number of other cell types undergo degeneration in *pcd* mice, including olfactory bulb mitral cells, retinal photoreceptor cells, and spermatocytes [1]. The mutation responsible for the *pcd* phenotype was mapped to the gene encoding cytosolic carboxypeptidase 1 (CCP1, also known as Nna1), and the gene was named *Agtpbp1* because the protein was initially considered to be an ATP/GTP binding protein [2]. CCP1 was discovered in a search for mRNAs upregulated in spinal motor neurons during regeneration after axotomy [3]. Thus, CCP1 is linked to both degeneration and regeneration. CCP1 has sequence homology to metallocarboxypeptidases, including conservation of critical active site residues, but lacks a signal peptide and is expressed in the cytosol [4]. Five additional members of the CCP1 subfamily were discovered and named CCP2 through CCP6 [4], [5]. CCP1 is the most abundant of the CCPs in mouse brain [4].

Altogether, nine independent phenotypic alleles of *pcd* have been characterized that are due to mutations in the *Agtpbp1* gene [6]–[8]. The *pcd3J* allele results from a deletion of exons 6–8, and the splicing of exon 5 to exon 9 introduces an inframe stop codon that results in a truncated protein lacking the active CP domain. The CP domain is critical for CCP1 function and normal mouse phenotype; expression of CCP1, but not CCP1 with mutated catalytic residues, in Purkinje cells of *pcd* mice prevents loss of these cells [9], [10]. Many studies have been done to characterize Purkinje cell death in mutant mice, and a number of potential mechanisms of neurodegeneration have been proposed [8], [11]–[15].

Based on the broad distribution of CCP1 mRNA in many tissues and cell types, and the expression of the protein in the cytosol, two potential functions for CCP1 were proposed. One is a role in protein/peptide turnover within a cell. Proteins are degraded by the proteasome into peptides, which need to be converted into amino acids and subsequently be recycled into proteins. Although evidence suggests that peptide degradation is largely carried out by aminopeptidases [16], [17], in theory it is possible that cytosolic carboxypeptidases also contribute to this process. Previously, some of the authors of the present study reported that adult *pcd* mouse brains have greatly elevated levels of peptides derived from intracellular proteins (i.e. those present in cytosol, mitochondria, and other non-secretory pathway compartments) [18]. This finding was interpreted as evidence that CCP1 participates in intracellular peptide degradation [18]. The other function proposed for CCP1 involved tubulin processing [4]. The alpha chain of tubulin undergoes trimming of the C-terminal Tyr and Glu residues. In addition, both alpha and beta tubulin are modified by addition of Glu or Gly to the gamma carboxyl group of a Glu located near the C-terminus, and these side chains are subsequently removed in a dynamic process [19]–[21]. Recently it was shown that CCP1 is capable of removing Glu residues from the C-terminus and polyglutamyl side chains of tubulin [22], [23]. Furthermore, the lack of CCP1 leads to tubulin hyperglutamylation. Knock-down or knock-out of tubulin tyrosine ligase-like-1, an enzyme that adds Glu to the side chain of tubulin, prevents neurodegeneration of Purkinje cells in *pcd* mice [22], [23]. Impaired tubulin polyglutamylation in other animal models is known to lead to mislocalization of molecular motors and affect tubulin-dependent trafficking and synaptic transmission [24]–[26].

To test if CCP1 functions in the degradation of intracellular peptides, we compared peptide levels in brains of presymptomatic *pcd* mice, before Purkinje cell death, to age-matched wild-type (WT) mice. We also measured relative peptide levels in non-neuronal tissues of adult *pcd* mice, selecting two organs (heart, spleen) which have high levels of CCP1 relative to other CCPs. Because protein turnover is a fundamental cellular process, if CCP1 participates in the degradation of proteasome-generated peptides, the absence of CCP1 activity in the *pcd* mice would be expected to produce a change in the intracellular peptidome. We also expanded our analysis of the adult *pcd* mouse brain by including peptides derived from secretory pathway proteins. The pattern of changes of peptides in adult mouse brain *pcd* mice was similar to the pattern found in mice lacking a gene necessary for the formation of primary cilia, a sensory cell organelle [27]. Because the disruption of primarily cilia has recently been shown to affect tubulin modifications [28], [29] and proteasome function [30], our finding further suggests that the altered peptidome in adult *pcd* mouse brain is a secondary change due to affected tubulin processing.

## Materials and Methods

### Animals

Animal use experiments were approved by the Institutional Animal Care and Use Committee of Albert Einstein College of Medicine (protocol #20090305). Multiple breeding pairs of *pcd* heterozygous mice (BALB/cByJ- Agtpbp1^pcd-3J^/J) were purchased from The Jackson Laboratory. Homozygous *pcd* mice and WT littermates were produced from matings of heterozygous *pcd* mice within the Animal Institute’s barrier facility at the Albert Einstein College of Medicine. The *Ift88* conditional cilia mutant mice (*Ift88^tm1.1Bky^*; hereafter called *ift88* cilia mutant mice) were described previously [31]. To delete cilia, six-eight week old female *ift88^f/f^* mice on the inducible CaGG-CreER (B6.Cg-Tg(CAG-cre/Esr1*)5Amc/J) background were injected with 6mg/40gm body weight tamoxifen suspended in corn oil for five consecutive days. Brains from the cilia mutant and control mice were collected at three months of age and processed for peptidomics analysis as described below. Cilia and IFT88 protein loss was confirmed by immunofluorescence microscopy in brain sections and by Western blot analysis on parallel sets of mice as described previously [32], [33].

### Quantitative Peptidomics

For the analysis of peptides in young amygdala and cerebellum, two WT and three *pcd* mice were used, each analyzed separately. All animals were 3-weeks old. For the analysis of peptides in spleen, three adult WT and two adult *pcd* mice were used. For the analysis of peptides in heart, two adult WT and three adult *pcd* mice were used. The analysis of peptides in adult brain was performed with two *pcd* and two WT mice. For the cilia mutant mice, brain peptides were analyzed in six cilia mutant and three control mice. For all peptidomics studies, tissues were heat-treated using microwave irradiation, as described [34]. Peptide extraction, labeling with isotopic tags, sample preparations, and peptide identification from tandem mass spectrometry (MS/MS) analysis were performed as described [18], [35]. Briefly, peptides were extracted with hot water and cold HCl, and labeled with the isotopic tags D0-, D3-, D6-, D9-, or D12-trimethylammonium butyrate (TMAB)-*N*-hydroxysuccinimide ester. After labeling, excess TMAB was quenched with Gly, the samples were pooled, filtered through YM-10 filters, treated with hydroxylamine to remove unspecific TMAB binding to Tyr residues, and desalted using C18 spin columns. Samples were analyzed by liquid chromatography/mass spectrometry (LC/MS) on a Waters Q-TOF–Ultima Mass Spectrometer or an API Q-Star Pulsar-i quadrupole time-of-flight mass spectrometer. MS/MS data analysis was performed using Mascot search engine (Matrix Science Ltd., U.K.). The IPI mouse database, which consists of 56,934 sequences; 25,565,245 residues, was searched. Methionine oxidation, N-terminal acetylation, and the isotopic D0-, D3-, D6-, and D9-TMAB tags were considered during peptide identification (D12-TMAB is not a search option on Mascot). The Mascot search results were manually interpreted using the following criteria: (1) The Mascot ion score was the top score of all analyzed peptides. However, this alone was insufficient to distinguish true positives from false positives, and all of the following additional criteria were also considered. (2) The D0-, D3-, D6-, or D9-isotopic form of TMAB identified by Mascot matched the isotopic form identified by analysis of the peak set. This criterion was important because Mascot does not discriminate between peak sets and cannot determine which of the peaks correspond to the D0, D3, D6, D9, or D12-labeled peptides. False positives have a 4 in 5 chance of having the wrong isotopic tag if one tag was incorporated, a 24 in 25 chance of having the wrong isotopic tags if two tags were incorporated, and a 124 in 125 chance of having the wrong isotopic tags if three tags were incorporated. Thus, this is an important criterion that excludes many false positives. (3) The number of labels incorporated into the peptide corresponded to the number of free amines (unmodified N terminus and side chains of Lys). If a peptide was labeled with several tags, then all tags represented the same isotopic form. (4) The major MS/MS fragment ions corresponded to predicted a, b, or y ions, internal ions, or precursor ions with loss of trimethylamine. (5) At least five fragment ions matched b or y ions. (6) The mass accuracy of the fragment ions was within 40 ppm, the accepted specification for the q-TOF instruments. (7) The observed charge state matched the predicted charge state based on the peptide sequence. (8) The fragment ions matched the expected ions based on the particular peptide sequence. For example, cleavage of Xaa-Pro bonds was favored and produced strong fragment ions while cleavage at Pro-Xaa bonds was rarely detected.

### Quantitative Real-time PCR

Total RNA was isolated from heart and spleen of adult WT and *pcd* mice using RNeasy Mini Kit (Qiagen). Superscript III first strand kit (Invitrogen) was used to synthesize cDNA from 2 µg of total RNA and random hexamers. Primers used for mouse CCP1 through CCP6 and GAPDH were previously described [23]. SYBR green fluorescent tag was introduced using Power SYBR Green PCR Master Mix (Applied Biosystems). PCRs were carried out on a 7900HT Real Time Thermal cycler (Applied Biosystems). All samples were run in triplicate. The threshold cycle number (Ct) was used to calculate quantitative values. The fold-change in expression was calculated using DDCt method.

### Immunohistochemistry

3-week-old WT and *pcd* mice were transcardially perfused with PBS and then with 4% paraformaldehyde (PFA). Tissue preparation and staining were performed as indicated [36]. Briefly, brains were fixed in 4% PFA overnight, incubated in 30% sucrose in PBS for 6 h, and frozen in optimal cutting temperature gel at −50°C. Cerebellum coronal sections (14 µm thick) were probed with anti-Calbidin-D28k (1∶3000, Sigma-Aldrich) followed by secondary Cy2-conjugated antibodies (Jackson Immunoresearch). The cerebellum sections were mounted in antifade reagent (Invitrogen) and then analyzed by fluorescence microscopy.

## Results

**Figure 1:**
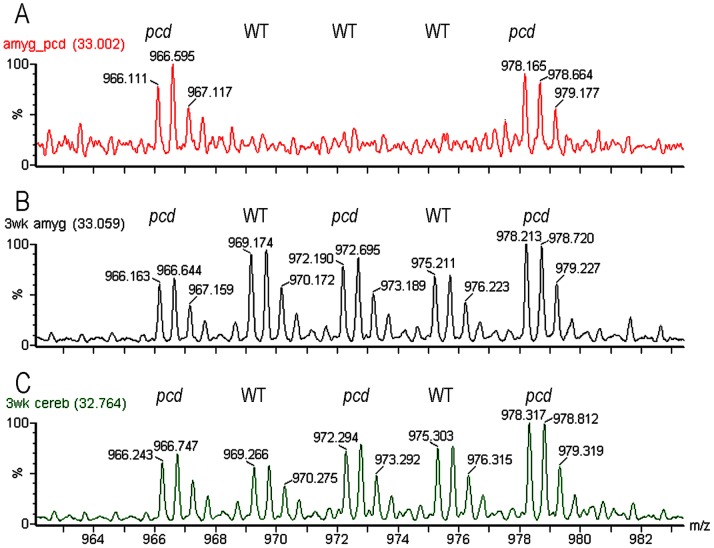
Representative data of quantitative peptidomics approach to measure changes in peptide levels in pcd mice, relative to WT mice. Representative spectra show the 2+ ions for the D0 to D12-TMAB peaks of the peptide subsequently identified by MS/MS as the N-terminal fragment of prefoldin 1, Ac-AASVDLELKKAFTEL, with TMAB tags on the two Lys residues. **A**, three different adult WT mice (labeled with D3-, D6-, and D9-TMAB) and two adult *pcd* mice (labeled with D0- and D12-TMAB). **B**, amygdala from 3 week old mice. **C**, cerebellum from 3 week old mice. For B and C, WT tissue was labeled with D3- and D9-TMAB, *pcd* tissue was labeled with D0-, D6-, and D12-TMAB.

**Figure 2:**
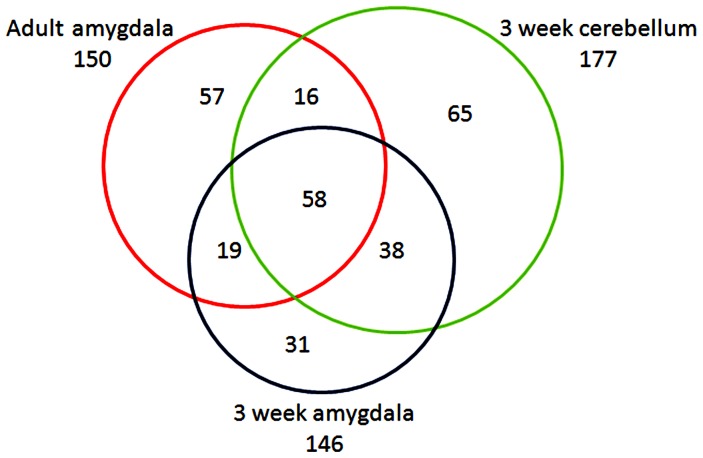
Overview of intracellular peptides identified in analysis of *pcd* and WT mice, comparing adult amygdala, 3 week old amygdala, and 3 week old cerebellum. For this analysis, the intracellular peptides listed in Table S1 as conclusively identified by MS/MS were considered; unknowns and tentatively identified peptides in Table S1 were not used for this analysis. Each peptide was counted once, regardless of whether it was detected in both *pcd* and WT mice or in only one of these genotypes. Peptides listed in Table S1 with multiple charge states were counted only once.

If CCP1 functions in peptide degradation in adult mouse brain, we reasoned that because protein/peptide degradation is such a fundamental process, CCP1 would also function in this capacity in young mouse brain, and therefore a peptidomics analysis of young *pcd* mouse brain would also show marked changes in levels of intracellular peptides. In contrast, if the previously observed increase in levels of many brain peptides was a consequence of the neurodegeneration in the adult *pcd* mice, then young mice analyzed prior to the onset of neurodegeneration would not show the increase in intracellular peptide levels. Purkinje cells start to degenerate in *pcd* mice when animals are 3 weeks old [1]; this was confirmed in our colony of *pcd* mice by immunohistochemistry (Figure S1). To test if young presymptomatic mice show greatly altered levels of the brain peptidome, a quantitative peptidomics approach involving TMAB isotopic labels was used to compare amygdala and cerebellum of 3 week old WT mice versus *pcd* mice. The five distinct masses of the TMAB labels allow 2–3 mutant mice to be compared to 2–3 WT mice in the same experiment; this allows for variation among replicates of WT animals to be determined along with the ratio of peptides in mutant versus WT mice. Representative MS data are shown in Figure 1, comparing relative levels of the same peptide in three different LC/MS runs. Panel A shows the LC/MS run for adult amygdala in which two *pcd* mice were compared to three WT mice. Panel B shows the LC/MS run for 3 week old amygdala in which three *pcd* mice were compared to two WT mice. Panel C shows the LC/MS run for 3 week old cerebellum in which three *pcd* mice were compared to two WT mice. For this peptide, levels are generally comparable between the *pcd* and WT mice when tested at 3 weeks of age, but clearly much higher in the adult *pcd* mice than the WT mice. Altogether, 146 distinct peptides arising from intracellular proteins were identified in the young amygdala and 177 distinct intracellular peptides identified in the young cerebellum; there was considerable overlap between the two sets of peptides (Figure 2). These two sets of peptides also showed considerable overlap with the intracellular peptides previously identified in the study comparing the amygdala of adult WT and *pcd* mice [36]. Data are provided in Table S1.

**Figure 3:**
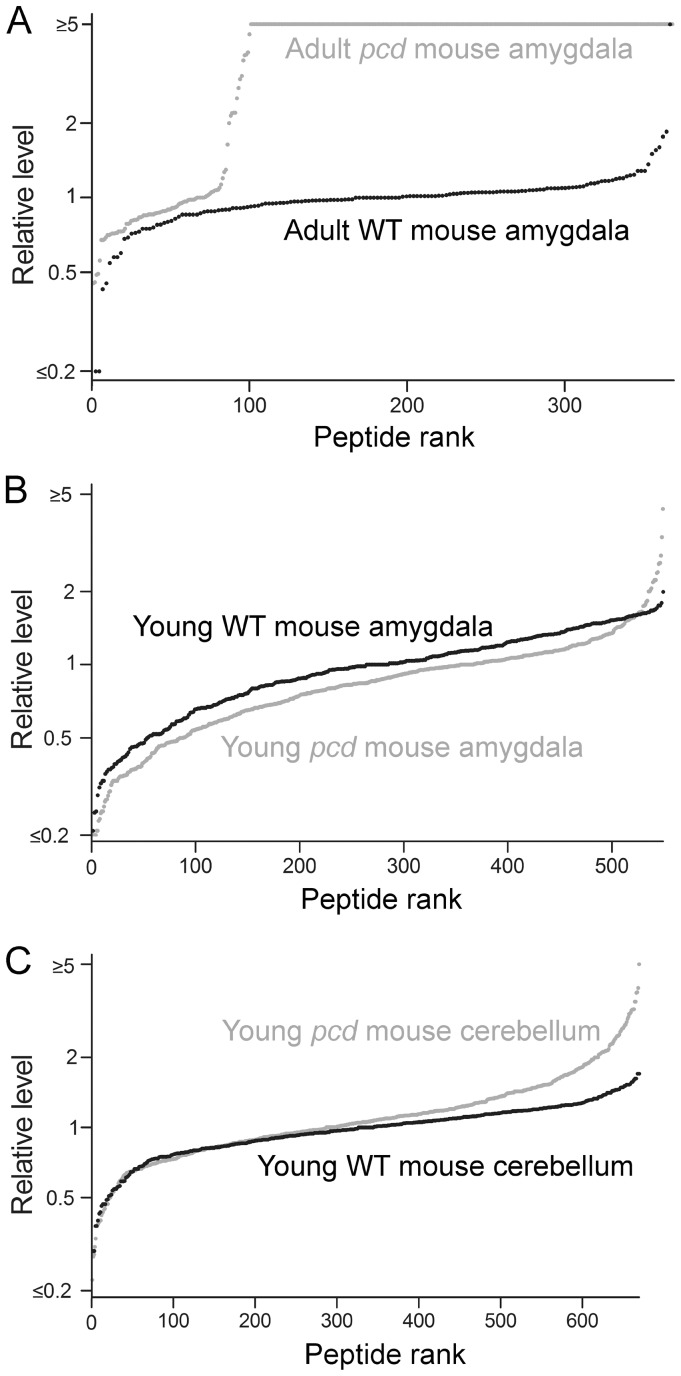
Levels of intracellular peptides in the brain of young WT and *pcd* mice. The levels of peptides derived from intracellular proteins were studied using quantitative peptidomics. The relative levels of peptides in WT mice (black circles) and *pcd* mice (grey circles) are indicated for (**A**) amygdala of adult mice; (**B**) amygdala of 3-week old mice; and (**C**) cerebellum of 3-week old mice. Each dot in the graph shows the ratio between a peptide in one WT or *pcd* replicate versus the average level in the WT replicates. The x-axis reflects the number of peptides found in *pcd* mice. See Table S1 for data.

A graphic representation of the data was prepared by plotting individual data points, sorted by the relative ratio of each intracellular peptide in a *pcd* mouse tissue, relative to the average level of that peptide in the same tissue of WT mice (Figure 3). For this analysis, each data point represents a peptide detected in one of the samples, the y-axis represents the ratio relative to the average level in the controls, and the x-axis represents the rank order of each data point (sorted from smallest ratio to largest ratio). This representation of the data shows a dramatic increase in the levels of most intracellular peptides previously identified in adult *pcd* mouse amygdala (Figure 3A, grey circles). For this analysis, the ratio of peptides seen only in the *pcd* mouse tissue and not in WT tissue was capped at 5, reflecting the typical signal-to-noise ratio of representative data (see Figure 1A). Conversely, peptides seen only in the WT tissue but not in the mutant tissue would be capped at a ratio of 0.2, also reflecting a 5-fold difference in the relative levels. The actual changes, increases or decreases, may be much larger than 5-fold, and this value is simply a conservative estimate based on the typical baseline signal. In figure 3A, it is clear that the majority of the identified peptides in the adult amygdala are like those in Figure 1A; detectable only in the *pcd* mice and not in the WT controls. For those peptides observed in WT animals, it was possible to determine the peptide level in each animal relative to the average WT value; this provides an estimate of mouse-to-mouse variation. Most of the peptides detected in WT mouse amygdala had ratios between 0.5 and 2.0 (Figure 3A, black circles). Contrary to the results with adult mouse amygdala, only a few of the intracellular peptides were greatly elevated in amygdala (Figure 3B, grey line) and cerebellum of young presymptomatic *pcd* mice (Figure 3C, grey line). Therefore, the absence of large changes in intracellular peptides in young *pcd* mouse brain regions suggests that accumulation of intracellular peptides later in life reflects secondary changes due to the CCP1 mutation in *pcd* mice.

**Figure 4:**
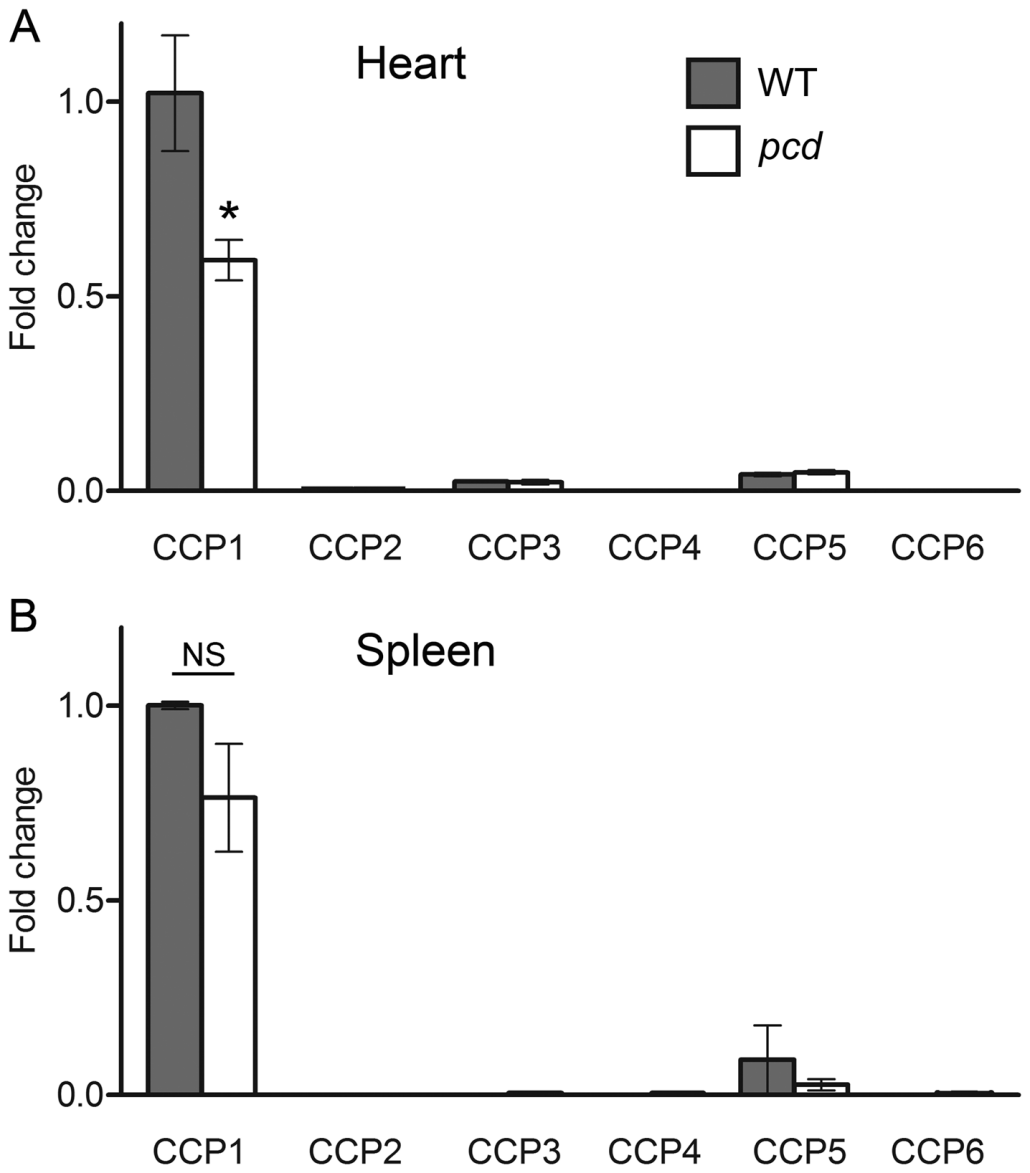
Relative levels of CCP mRNAs in selected tissues of WT and *pcd* mice. mRNA levels of CCP1 through 6 in heart and spleen (panels **A** and **B**, respectively) of adult WT and mutant mice were analyzed using quantitative real-time PCR. CCP1 mRNA is the most abundant transcript in the heart and spleen of WT animals. The levels of CCP1 transcript are decreased in *pcd* mice, but the levels of other CCP transcripts are not affected. Fold change in expression was calculated using the DDCt method. GAPDH was used as an internal control. n = 3 for each genotype. *, p<0.05 using Student’s t-test. NS, not significant.

In addition to examining levels of intracellular peptides in young brain tissue, we also examined peptides in adult mouse brain and other adult tissues. Before performing peptidomic analyses, we first needed to identify appropriate tissues for this analysis. It is important to consider levels of CCP2-6 because these other enzymes can potentially compensate for the absence of CCP1 in *pcd* mice. Levels of CCP1 mRNA in WT and *pcd* brain are higher than levels of mRNA for CCP2-6 based on *in situ* hybridization and reverse transcription PCR [4]. This is also supported by bioinformatic analysis of expressed sequence tag (EST) databases (http://www.ncbi.nlm.nih.gov/UniGene/). In addition to brain, many other tissues show relatively high levels of CCP1 based on PCR analysis and bioinformatics analysis of EST databases. For example, heart and spleen have levels of CCP1 mRNA generally comparable to brain [4]. Heart and spleen have much lower levels of CCP2-6 mRNA, based on previous PCR results [4] and EST database analysis. To confirm, and to determine if the other CCP mRNAs are up-regulated in *pcd* mice, we performed QRT-PCR on the heart and spleen of adult WT and *pcd* mice. As predicted from EST database analysis, CCP1 mRNA is reasonably abundant in WT mouse heart and spleen (Figure 4A and B, respectively). The relative levels of all other CCP mRNAs in these organs are much lower than CCP1 mRNA (Figure 4). The level of CCP1 transcript in heart is lower in *pcd* compared to WT mice (Figure 4). The levels of other CCP mRNAs are not significantly changed in *pcd* mice, suggesting that there is no compensation for the absence of enzymatically active CCP1 in mutant animals.

**Figure 5:**
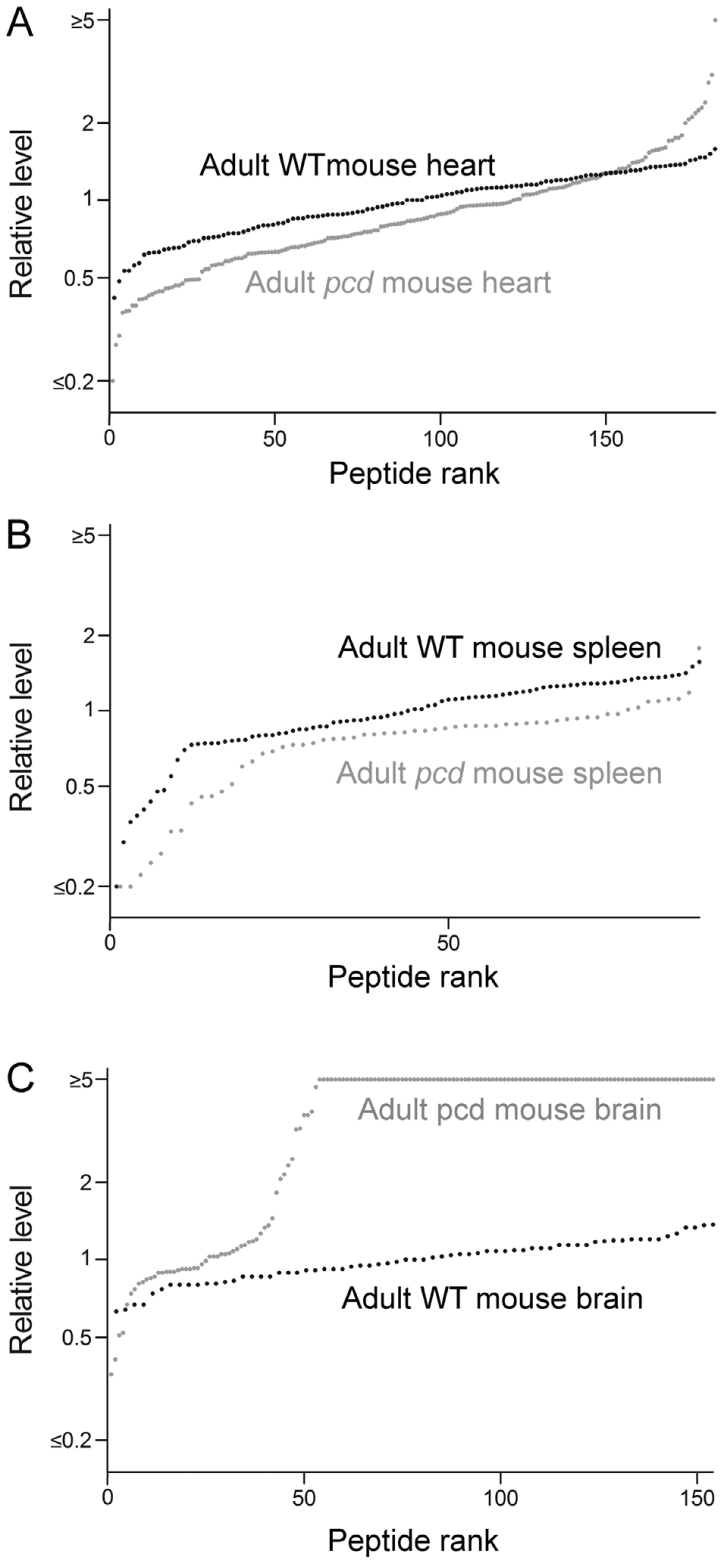
Levels of intracellular peptides in organs of adult WT and *pcd* mice. The levels of peptides derived from cytosolic, mitochondrial, and nuclear proteins were analyzed using quantitative peptidomics. Each dot in the graph shows the ratio between a peptide in one WT or *pcd* replicate versus the average level in the WT replicates. The y-axis is logarithmic and shows the relative levels of peptides of adult WT mice (black circles) and adult *pcd* mice (grey circles) for (**A**) heart, (**B**) spleen, and (**C**) brain lacking the olfactory bulb and cerebellum. The x-axis indicates the relative rank order of each peptide. The x-axis reflects the number of peptides found in *pcd* mice. See Table S1 for data.

Peptide levels in adult *pcd* mouse heart, spleen, and brain were compared to levels in WT mice. Data are included in Table S1, and graphic representations shown in Figure 5. Levels of peptides in *pcd* heart and spleen are generally comparable to the levels of peptides in the corresponding WT mouse tissues (Figure 5A and B). In contrast, the majority of peptides derived from cytosolic and mitochondrial proteins observed in the *pcd* mouse brain are not detectable in control mouse brain, defined as a *pcd*/WT ratio >5 (Figure 5C). For the analysis in Figure 5C, adult mouse brains lacking the cerebellum and olfactory bulb were used in order to avoid potential problems due to cell death that occurs in these brain regions. The changes in peptide levels observed in adult *pcd* mouse brains in the present study were similar to the changes previously reported when levels of intracellular peptides were analyzed in adult *pcd* mouse brain hypothalamus, amygdala, cortex, striatum, and hippocampus [18]. Thus, including the two additional *pcd* mice analyzed in the present study, the dramatic increase in levels of intracellular peptides in the brain of adult mice was detected in 8 distinct *pcd* mice, compared to 11 distinct WT mice.

**Figure 6:**
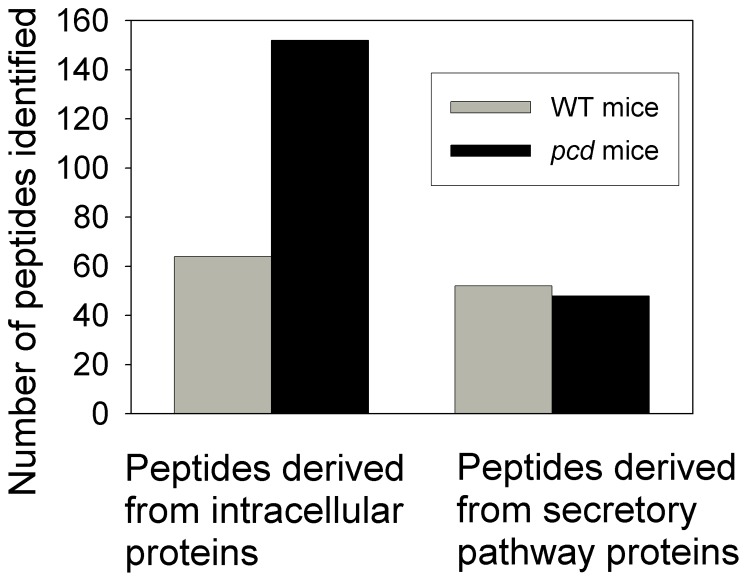
Comparison of the number of peptides detected in adult *pcd* and WT mouse brain. Data from the analysis of peptides in whole brain (minus the cerebellum and olfactory bulb) from adult mice indicated in Table S1 were used. For this study, two WT mice were compared to two *pcd* mice. Conclusively and tentatively identified peptides were included in this analysis, and the peptides were counted each time they were detected as a separate m/z ion (i.e. for each animal, and for each charge state).

**Figure 7:**
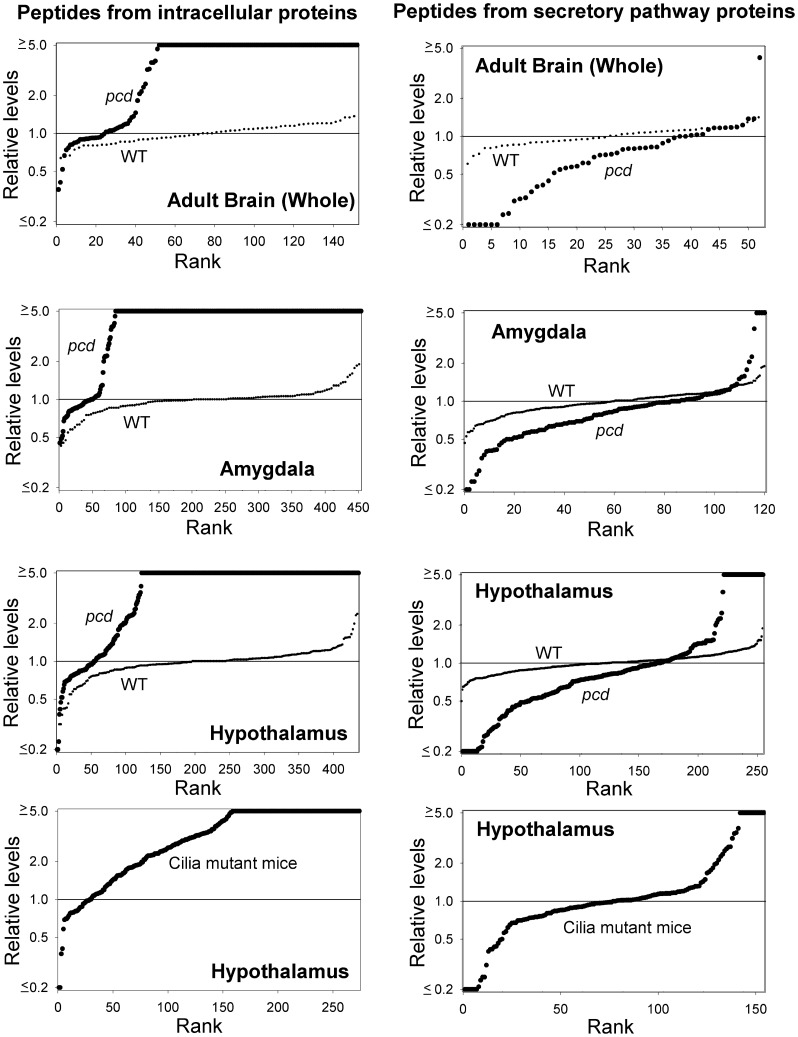
Relative levels of intracellular and secretory pathway peptides in adult WT and mutant mice. Peptides identified in adult whole brain (minus the cerebellum and olfactory bulb) or in amygdala or hypothalamus were included in this analysis. For the top three panels, each dot in the graph shows the ratio between a peptide in one WT or *pcd* mouse versus the average level in the WT replicates. Large black circles represent *pcd* mice, small black circles represent WT mice. The x-axis reflects the number of peptides found in *pcd* mice. For the bottom panels, the data represent an earlier study that used only two isotopic TMAB labels (D0 and D9). For these experiments, one group of cilia mutant mice were labeled with D0 and the WT mice controls with D9, while another group of cilia mutant mice were labeled with D9 and WT controls with D0, and for both LC/MS runs the peptide levels in cilia mutant mice were compared to the WT controls in the same LC/MS run. Because of this labeling strategy, it was not possible to compare WT to WT mice for these data because the different groups of WT mice were in separate LC/MS runs and were not directly comparable (in contrast to the studies on *pcd* mice which used 4–5 isotopic labels and allowed for the different WT replicates to be analyzed in the same LC/MS run). See Table S1 for data.

Altogether, 152 peptides derived from intracellular proteins were identified in the new analysis of the adult *pcd* mouse brain, whereas only 64 peptides derived from intracellular proteins were identified in the WT mice included in the LC/MS analysis (Figure 6). In addition to analyzing peptides derived from intracellular proteins, the new analysis of adult *pcd* and WT mouse brain considered all peptides detected in the sample, which included peptides derived from secretory pathway proteins (i.e. neuropeptides and related molecules). A total of 52 secretory pathway peptides were found in the WT brains and 48 were found in the *pcd* brains (Figure 6). However, when the relative levels of secretory pathway peptides were plotted on rank plots, there was a clear decrease in levels of these peptides in the *pcd* mice (Figure 7, top right panel). For comparison, the rank plot of the peptides from intracellular proteins previously shown in Figure 5C is included in Figure 7 (top left panel). To test if the decrease in secretory pathway peptides was also observed in the data on the adult amygdala and hypothalamus (which were derived from different groups of animals than the study on whole brain), we reanalyzed the previous data for peptides derived from secretory pathway proteins. As found for whole brain, some of the secretory pathway peptides detected in amygdala and hypothalamus showed a decrease in *pcd* mice, relative to WT mice (Figure 7, right panels). However, some secretory pathway peptides also showed a large increase in *pcd* mouse amygdala and hypothalamus (Figure 7). The identity of these peptides and relative levels are included in Table S1, and representative peptides highlighted in the next two figures described below.

The quantitative peptidomics approach used in the present study has previously been used for the analysis of brain peptides in other mutant mice, and with one exception (described below), the pattern of changes in intracellular and secretory pathway peptides of adult *pcd* mouse brain was unique. Some of the mutants analyzed showed large changes in levels of secretory pathway peptides, but no large changes in levels of most intracellular peptides (Figure S2). These mutants include mice lacking carboxypeptidase E (CPE) activity (*Cpe^fat/fat^* mice) and mice with targeted disruptions of the genes encoding prohormone convertase 2 [37]–[39]. Because these enzymes function in the biosynthesis of neuropeptides, the change in levels of secretory pathway peptides was expected in these mutant mice. Other mutant mice lines that have been examined include mice with disruptions in the genes encoding prohormone convertase 1 (also known as prohormone convertase 3), proprotein convertase 7, endothelin converting enzyme 2, and *ift88*. Prohormone convertase 1/3, proprotein convertase 7, and endothelin converting enzyme 2 are peptidases in the secretory pathway while Ift88 is a protein required for intraflagellar transport and the formation of cilia [40]–[45]. In most of these previous studies, the relative levels of peptides derived from intracellular proteins were generally similar between mutant and WT mice. However, there was one exception; Ift88 cilia mutant mice showed a dramatic increase in the levels of the majority of hypothalamic peptides derived from intracellular proteins (Figure 7, lower left panel). Analysis of striatal and thalamic peptides showed a similar effect in the Ift88 cilia mutant mice (Table S1). Interestingly, hypothalamic peptides derived from secretory pathway proteins were also found to be altered in this mouse mutant, with some peptides elevated and other decreased, relative to WT control mice (Figure 7, lower right).

**Figure 8:**
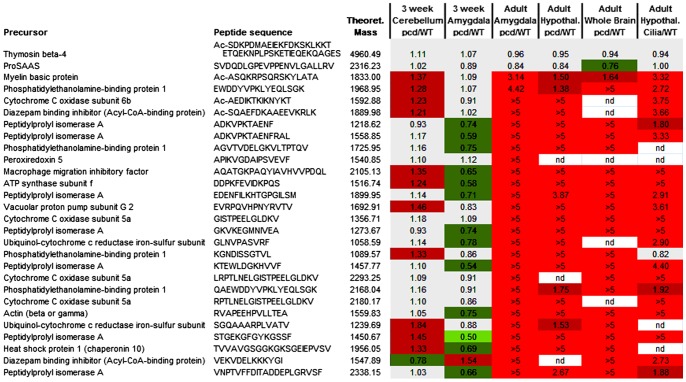
Heat map representation of relative levels of intracellular peptides. Peptides derived from intracellular proteins that were commonly detected in young and adult mouse brain were included in this analysis. Each row represents a different peptide. The color shows the ratio between a peptide in one WT or mutant mouse replicate versus the average level in WT mice within each LC/MS run. Bright green represents neuropeptides which are not detectable in a mutant mouse or present at low levels (ratio less than 0.50). Dark green indicates peptides which are slightly reduced in a mutant mouse (ratio 0.51 to 0.80). Grey shows peptides with a ratio between 0.81 and 1.19. Dark red represents slightly increased in a mutant mouse (ratio 1.20 to 1.99), and bright red shows peptides greatly increased in a mutant mouse (ratio >2.0). Nd, not detected. See Table S1 for data.

**Figure 9:**
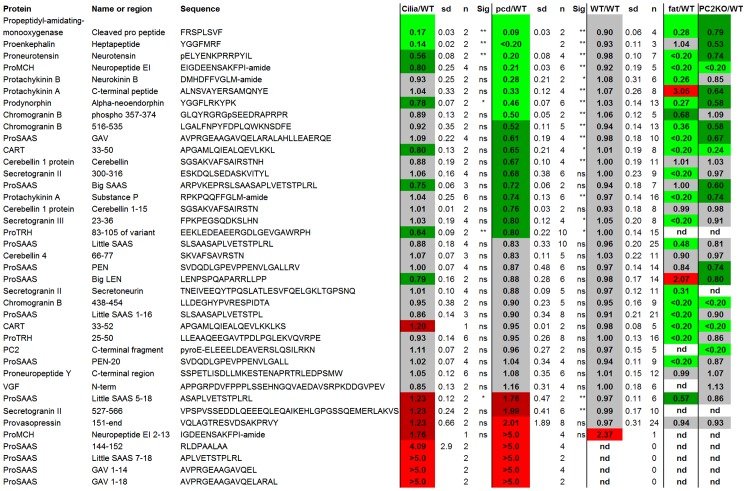
Heat map representation of relative levels of secretory pathway peptides in adult *pcd* and other mutant mouse brains. Peptides derived from secretory pathway proteins that were commonly detected in the hypothalamus of cilia mutants, *pcd*, WT, *Cpe^fat/fat^,* and prohormone convertase 2 knockout mice were included in this analysis. Each row represents a different peptide. The color shows the ratio between a peptide in one WT or mutant mouse replicate versus the average level in WT mice within each LC/MS run. The color scheme is identical to that used in Figure 8. Additional abbreviations: sd, standard deviation; n, number of replicates; sig, statistical significance of difference with the WT/WT value (calculated using Student’s t-test); *, p<0.05; **, p<0.01; ns, not significant; fat/WT, the ratio of peptides in *Cpe^fat/fat^* mice relative to WT mice; PC2KO/WT, the ratio of peptides in prohormone convertase 2 knock-out mice relative to WT mice. See Table S1 for data.

Although the rank plots shown in Figure 7 (and other figures) are useful to compare the overall peptidome between different groups of mice, these plots do not convey information about the individual peptides. In order to compare individual peptides, we used heat maps in which peptides commonly found in the various analyses were selected and the average ratio of peptide in mutant mice, relative to WT mice, was calculated. Then, the value was color-coded, with bright green representing large decreases (ratio of mutant to WT ≤0.50), dark green representing small decreases (ratio 0.51 to 0.80), grey representing no substantial changes (ratio 0.81 to 1.19), dark red representing small increases (ratio 1.20 to 1.99) and bright red representing large increases (ratio ≥2.0). This analysis was performed for peptides derived from intracellular proteins (Figure 8) and also for peptides derived from secretory pathway proteins (Figure 9). For the intracellular peptides, many of the peptides found to increase in the adult whole brain were also found to increase in the adult amygdala and cerebellum (Figure 8). Only small changes were found in peptides in the 3 week old *pcd* mouse cerebellum and amygdala, consistent with the rank plots shown in other figures (Figure 8). Interestingly, the Ift88 cilia mutant mice showed changes in many of the same intracellular peptides as observed in the adult *pcd* mice (Figure 8).

Heat map analysis of the secretory pathway peptides was performed, and because many of these peptides were detected in WT mice, it was possible to compare levels using statistical tests. Of the 8 peptides that decreased substantially in the *pcd* mice that were also found in the other experiments, 4 of these were significantly decreased in the Ift88 cilia mutant mice (Figure 9). Of the 16 peptides that were not significantly altered in the *pcd* mice, all 16 were not significantly affected in the Ift88 cilia mutant mice either. Furthermore, the 8 peptides elevated in the *pcd* mice were also found to be elevated in the Ift88 cilia mutant mice, although for these peptides statistical testing of the changes between mutant and WT mice could not be performed (statistical testing requires a number, and a ratio of >5 is not a number). In contrast to the general similarity between the changes in secretory pathway peptides between *pcd* mice and Ift88 cilia mutant mice, there was no obvious correlation between either of these mutants and the *Cpe^fat/fat^* mouse or prohormone convertase 2 KO mouse (Figure 9).

**Figure 10:**
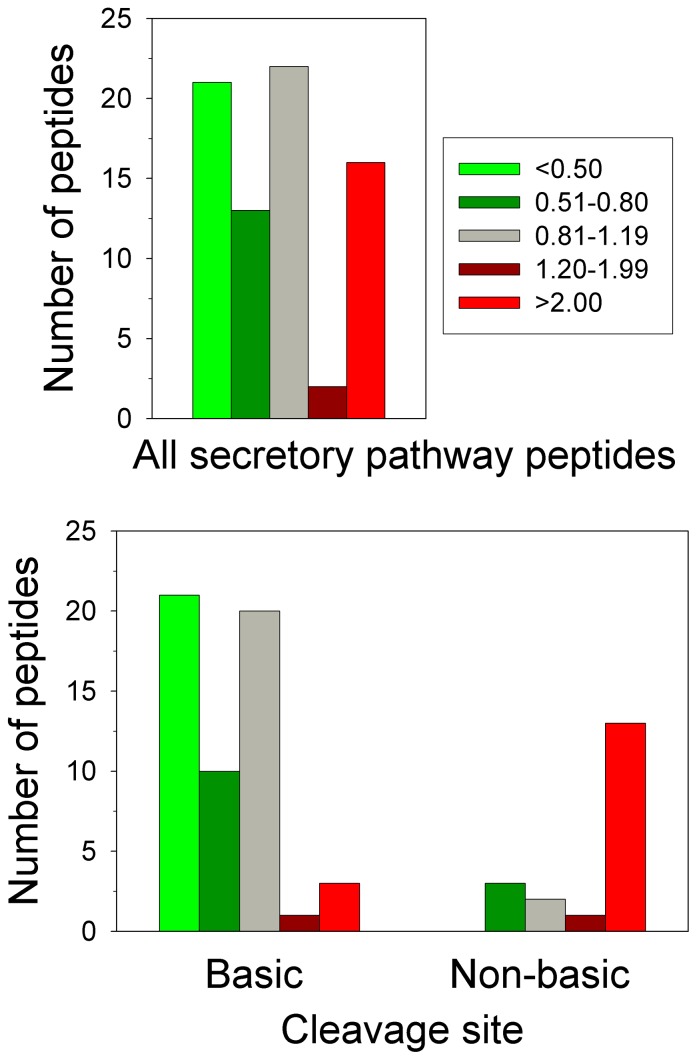
Analysis of peptides derived from secretory pathway proteins. Secretory pathway peptides detected in *pcd* mouse amygdala and hypothalamus were divided into the same five groups as described in Figure 8. The y-axes indicate the number of peptides in all LC/MS runs. **Lower panel**: Peptides were divided into two categories based on the cleavage sites required to release the peptide from the precursor, and plotted as above. Left: peptides generated by cleavage at prohormone convertase consensus sites and/or other sites containing basic amino acids (lysine, arginine). Right: peptides generated by cleavage at a non-basic site at either the N- or C-terminal side of the peptide.

The secretory pathway peptides were analyzed further in an attempt to understand why some peptides increased while others decreased in the adult *pcd* mouse brain. For this analysis, the ratio of results from all secretory pathway peptides identified in amygdala, hypothalamus, and/or whole brain were combined to provide a larger number of peptides, and the changes in peptide levels were averaged so that for each peptide a single value was used. Then, the ratio between peptide levels in the *pcd* mouse and the levels of the corresponding peptide in WT mice within each LC/MS run was used to divide the peptides into five groups, as used for the heat map analysis. Similar to the heat map shown in Figure 9, which focused on only those peptides found in multiple experiments, the analysis of all identified peptides showed relative few peptides in the “slight increase” category (Figure 10, dark red bars), and more peptides in the other 4 categories. Analysis of the C-terminal amino acid of the peptides in each group did not reveal any obvious differences (data not shown). However, consideration of the cleavage site(s) required to generate the peptide revealed that most of the peptides that were substantially decreased in adult *pcd* mice arose from cleavage at prohormone convertase 1/3 and/or prohormone convertase 2 consensus sites (i.e. RR, KR, RxxR, and related sequences) or at a site containing a basic residue other than a prohormone convertase consensus site (Figure 10, Lower panel). Many of these peptides are known neuropeptides that represent the mature form present within large dense-core secretory granules. The majority of unchanged peptides were also in this group (Figure 10, Lower panel). Nearly all of the peptides that increased in adult *pcd* mice were produced by processing at a non-basic site on either the N- or C-terminal side of the peptide (Figure 10, Lower panel). Many of these cleavages may represent extracellular cleavage by peptidases present in the synapse or lysosomal enzymes after fusion of lysosomes with vesicles.

## Discussion

The overall goal of the present study was to determine whether CCP1 plays a role in peptide turnover within the cell. Previously, some of the authors of the present study proposed that CCP1 contributed to peptide degradation based on the dramatic increase in levels of intracellular peptides observed in *pcd* mice [18]. While these authors had proposed that CCP1 could also play a role in tubulin processing, the simplest explanation to account for the increase in peptides derived from intracellular proteins was that these peptides represented substrates of CCP1. This was found to be the case for peptides that accumulate in the brains of *Cpe^fat/fat^* mice, which lack carboxypeptidase E activity due to a point mutation [46]. However, the *Cpe^fat/fat^* mice showed both an increase in levels of substrates and a decrease in levels of products, as expected for a normal substrate-product relationship [47]. In contrast, the adult *pcd* mouse brain peptides were mainly elevated, which was interpreted to indicate that CCP1 degraded all peptides that it came into contact with [18]. However, the peptides elevated in adult *pcd* mouse brains contain a wide range of C-terminal amino acids; if these peptides represent substrates, then CCP1 has a very broad substrate specificity. This was a surprising observation; even the metallocarboxypeptidases present in the digestive system show marked preference towards particular amino acids [48]–[52]. Additional problems with this hypothesized function for CCP1 were found from studies overexpressing and knocking down the protein in HEK293 cells; only a small number of peptides were significantly elevated by the knock-down of CCP1, and no peptides were significantly altered by the overexpression of the protein [23]. Because protein turnover is a fundamental process that occurs in every cell of the body, it was expected that if CCP1 performs this role in adult mouse brain, it would also perform this role in cultured cells as well as in young mouse brain and other tissues. Therefore, the goal of the present study was to address the function of CCP1 by examining peptide levels in *pcd* mouse tissues that do not undergo degeneration, and young brain, prior to the degeneration of Purkinje cells.

Our present studies show that accumulation of peptides does not occur in the brain of young *pcd* mice and in heart and spleen of adult mutants. Since the adult mouse heart and spleen express high levels of CCP1 mRNA and low levels of other CCPs, it was expected that the lack of CCP1 in *pcd* mice would cause the same changes as in adult *pcd* mouse brain. It is conceivable that the normal peptide profile in *pcd* heart and spleen is achieved through a compensatory mechanism, although our results demonstrate that levels of mRNA encoding other CCPs are not upregulated by the absence of CCP1 in adult *pcd* mice. The finding that intracellular peptides are unchanged in the brain of young *pcd* mice also complicates the hypothesis that CCP1 functions in peptide turnover. Unless peptide turnover proceeds by a different mechanism in adult brain versus young mouse brain, adult spleen, and adult heart, these results suggest that the previous hypothesized role for CCP1 in peptide turnover is not correct.

The other hypothesized role for CCP1 involved the processing of tubulin, which undergoes a series of C-terminal reactions. For example, the C-terminal Tyr and Glu of alpha-tubulin are removed to form delta-2 tubulin. In addition, both alpha- and beta-tubulin can be modified by the attachment of Gly or Glu residues to the gamma-carboxyl group of Glu residues near the C-terminus of the protein, and these polyGlu or polyGly chains are removed by a carboxypeptidase. Several recent studies have shown that CCP1 is able to process tubulin, specifically removing Glu from the C-terminus to generate delta-2 tubulin, and also removing Glu from the side chains near the C-terminus of tubulin [22], [23]. Because CCP1 does not cleave any residue other than Glu from tubulin, it is further unlikely that this enzyme is a general degradative enzyme that cleaves a wide range of amino acids from peptides. Brain tubulin is more heavily glutamylated than tubulin in other organs, and *pcd* mice show hyperglutamylation of tubulin [23]. The present findings that peptides are affected in adult mouse brain but not other organs correlates with the hyperglutamylation of tubulin. Furthermore, the formation of delta-2 tubulin in brain is age-dependent, with young mice showing much lower levels of this form [53]. Thus, the age-dependence of the peptide accumulation in the brains of *pcd* mice also correlates with the age-dependence of the appearance of delta-2 tubulin.

Although initially unexpected, our observation that the peptidome of adult *pcd* mouse brain has many similarities to that of the cilia mutant animals also fits with a role for CCP1 in the modification of tubulin. Recently, it was found that tubulin is hyperacetylated in a kidney cell line that lacks primary cilia due to the absence of Kif3a expression [54]. Impaired tubulin polyglutamylation causes cilia dysfunction which leads to primary ciliary dyskinesia [55] and affected development of the spermatid flagella in mice [56]. Recently, it was reported that hyper-glutamylation of β-tubulin destabilizes ciliary microtubules in *Tetrahymena* [57]. Moreover, it was shown that CCP1 down-regulation causes cilia shortening in cell culture [58]. In addition, it was demonstrated that CCP1 regulates the ciliary localization and velocity of molecular motors, and loss of CCP1 affects cilia stability but not ciliogenesis in *C. elegans* [59]. Very recently, CCPs were linked to cilia using a bioinformatics approach [60]. Tubulin within the axoneme of cilia is highly glutamylated, and the levels of this modification decrease as the cilia mature [61]. Thus, the deglutamylating activity of CCP1 may not be required until later in life when cilia are formed. In agreement with this, it was shown that ciliogenesis is not affected in *C. elegans* CCP1 mutant, but ciliary defects develop gradually in an age-dependant manner [59].

While it is clear that CCP1 is involved in tubulin processing, and this can potentially account for the similar peptidome changes in cilia mutant and *pcd* mouse brain peptides, the mechanism by which altered tubulin processing affects the brain peptidome is less clear. The changes in secretory pathway peptides could potentially result from altered trafficking and/or storage of the peptide-containing secretory granules. It has been shown that impaired polyglutamylation of tubulin affects synaptic transmission [25]. The changes in secretory pathway peptides observed in our data are consistent with elevated secretion of peptides accompanied by extracellular processing. This would account for the reduced levels of many mature neuropeptides and the increased levels of peptides that are produced by processing at non-basic sites. For example, the melanin concentrating hormone peptide, known as peptide EI, is greatly reduced in the *pcd* mice while the shorter form of this peptide lacking the N-terminal residue is greatly elevated in *pcd* mice (Table S1). Interestingly, peptides from broadly expressed secretory pathway proteins such as chromogranin B and proSAAS represent many of those peptides unchanged in the *pcd* mice while peptides from prohormones expressed in more limited cell types such as protachykinin A, proenkephalin, prodynorphin, proopiomelanocortin, and promelanin concentrating hormone all have several peptides that are greatly decreased in the *pcd* mouse. This suggests that the changes in secretory pathway peptide levels in the *pcd* mouse are more pronounced in selected neurons.

The mechanism by which levels of peptides derived from cytosolic and mitochondrial proteins are altered by changes in tubulin processing is less clear. These peptides are presumably generated by the proteasome, based on recent findings that the vast majority of peptides present in human cell lines (HEK293 and SH-SY5Y) are affected by the proteasome inhibitors epoxomicin [62] and bortezomib [63]. Although this has not been examined yet for brain peptides, many of the peptides found in mouse brain are identical to those found in the cell lines [64], [65], and therefore it is likely that the brain peptides are also generated by the proteasome. If so, then the observed results could be explained by an effect of tubulin modification on proteasome activity, either directly or indirectly. Knockdown of proteins essential for the formation of the basal body influences proteasome function, leading to altered protein turnover [30]. The altered levels of intracellular peptides in cilia mutant mice observed in the present study is consistent with altered proteasome function. Taken together, one possible explanation of the altered levels of intracellular peptides in adult *pcd* mouse brain is that the defect in CCP1 leads to altered ciliary function which in turn affects proteasome activity and peptide levels. Alternatively, the defect in CCP1 activity in the adult *pcd* mouse brain may lead directly to changes in proteasome function and peptide levels, independent of the effect of CCP1 on ciliary function. While many details of the precise mechanism by which CCP1 defects lead to altered peptide levels in adult mouse brain, the original model that CCP1 plays a direct role in the degradation of intracellular peptides is not supported by the data reported in the present paper or by other recent results showing CCP1 selectively cleaves Glu from tubulin.

## Supporting Information

Figure S1 **Distribution of calbindin staining in cerebellum of 3-week-old WT and** ***pcd*** **mice.** WT and *pcd* cerebellar sections were probed with antibodies against calbindin, a marker for Purkinje cells. Staining shows Purkinje cell dendrites, Purkinje cell bodies, and Purkinje cell axons (all green) in WT (left panel) and *pcd* (right panel) cerebellum.
(TIF)

Figure S2 **Relative levels of intracellular and secretory pathway peptides in the hypothalamus of adult mice.** Data were plotted as described for Figures 3, 5, and 7. All values represent the ratio of the level of a particular peptide in the mutant mouse hypothalamic extracts, relative to WT mouse hypothalamic extracts. **A**,Intracellular peptides from *Cpe^fat/fat^* mice. **B**, Secretory pathway peptides from *Cpe^fat/fat^* mice. **C**, Intracellular peptides from prohormone convertase 2 KO mice, **D**, Secretory pathway peptides from prohormone convertase KO mice.
(TIF)

Table S1 **Data for individual peptides found in WT,** ***pcd*** **, and other mutant mice discussed in the paper.** Peptide sequences are indicated along with information on the protein precursor (name, subcellular location). The ratio of peptide levels in mutant versus WT tissue are indicated for each biological replicate. Different worksheets in the table correspond to the various mutant mouse studies.
(XLSX)

## References

[1] Mullen RJ, Eicher EM, Sidman RL (1976) Purkinje cell degeneration, a new neurological mutation in the mouse. Proc Natl Acad Sci USA 73: 208–212. 1061118 10.1073/pnas.73.1.208 PMC335870

[2] Fernandez-Gonzalez A, La Spada AR, Treadaway J, Higdon JC, Harris BS, et al (2002) Purkinje cell degeneration (pcd) phenotypes caused by mutations in the axotomy-induced gene, Nna1. Science 295: 1904–1906. 11884758 10.1126/science.1068912

[3] Harris A, Morgan JI, Pecot M, Soumare A, Osborne A, et al (2000) Regenerating motor neurons express Nna1, a novel ATP/GTP-binding protein related to zinc carboxypeptidases. Mol Cell Neurosci 16: 578–596. 11083920 10.1006/mcne.2000.0900

[4] Kalinina E, Biswas R, Berezniuk I, Hermoso A, Aviles FX, et al (2007) A novel subfamily of mouse cytosolic carboxypeptidases. FASEB J 21: 836–850. 17244818 10.1096/fj.06-7329com

[5] Rodriguez de la Vega M, Sevilla RG, Hermoso A, Lorenzo J, Tanco S, et al (2007) Nna1-like proteins are active metallocarboxypeptidases of a new and diverse M14 subfamily. FASEB J 21: 851–865. 17244817 10.1096/fj.06-7330com

[6] Chakrabarti L, Neal JT, Miles M, Martinez RA, Smith AC, et al (2006) The Purkinje cell degeneration 5J mutation is a single amino acid insertion that destabilizes Nna1 protein. Mamm Genome 17: 103–110. 16465590 10.1007/s00335-005-0096-x

[7] Wang T, Morgan JI (2007) The Purkinje cell degeneration (pcd) mouse: an unexpected molecular link between neuronal degeneration and regeneration. Brain Res 1140: 26–40. 16942761 10.1016/j.brainres.2006.07.065

[8] Li J, Gu X, Ma Y, Calicchio ML, Kong D, et al (2010) Nna1 mediates Purkinje cell dendritic development via lysyl oxidase propeptide and NF-kappaB signaling. Neuron 68: 45–60. 20920790 10.1016/j.neuron.2010.08.013 PMC4457472

[9] Wang T, Parris J, Li L, Morgan JI (2006) The carboxypeptidase-like substrate-binding site in Nna1 is essential for the rescue of the Purkinje cell degeneration (pcd) phenotype. Mol Cell Neurosci 33: 200–213. 16952463 10.1016/j.mcn.2006.07.009

[10] Chakrabarti L, Eng J, Martinez RA, Jackson S, Huang J, et al (2008) The zinc-binding domain of Nna1 is required to prevent retinal photoreceptor loss and cerebellar ataxia in Purkinje cell degeneration (pcd) mice. Vision Res 48: 1999–2005. 18602413 10.1016/j.visres.2008.05.026 PMC2602839

[11] Baurle J, Grusser-Cornehls U (1994) Axonal torpedoes in cerebellar Purkinje cells of two normal mouse strains during aging. Acta Neuropathol 88: 237–245. 7810294 10.1007/BF00293399

[12] Kyuhou S, Kato N, Gemba H (2006) Emergence of endoplasmic reticulum stress and activated microglia in Purkinje cell degeneration mice. Neurosci Lett 396: 91–96. 16356646 10.1016/j.neulet.2005.11.023

[13] Chakrabarti L, Eng J, Ivanov N, Garden GA, La Spada AR (2009) Autophagy activation and enhanced mitophagy characterize the Purkinje cells of pcd mice prior to neuronal death. Mol Brain 2: 24. 19640278 10.1186/1756-6606-2-24 PMC2729476

[14] Chakrabarti L, Zahra R, Jackson SM, Kazemi-Esfarjani P, Sopher BL, et al (2010) Mitochondrial dysfunction in NnaD mutant flies and Purkinje cell degeneration mice reveals a role for Nna proteins in neuronal bioenergetics. Neuron 66: 835–847. 20620870 10.1016/j.neuron.2010.05.024 PMC3101252

[15] Baltanas FC, Casafont I, Lafarga V, Weruaga E, Alonso JR, et al (2011) Purkinje Cell Degeneration in pcd Mice Reveals Large Scale Chromatin Reorganization and Gene Silencing Linked to Defective DNA Repair. J Biol Chem 286: 28287–28302. 21700704 10.1074/jbc.M111.246041 PMC3151073

[16] Goldberg AL, Cascio P, Saric T, Rock KL (2002) The importance of the proteasome and subsequent proteolytic steps in the generation of antigenic peptides. Mol Immunol 39: 147–164. 12200047 10.1016/s0161-5890(02)00098-6

[17] Reits E, Griekspoor A, Neijssen J, Groothuis T, Jalink K, et al (2003) Peptide diffusion, protection, and degradation in nuclear and cytoplasmic compartments before antigen presentation by MHC class I. Immunity. 18: 97–108. 10.1016/s1074-7613(02)00511-3 12530979

[18] Berezniuk I, Sironi J, Callaway MB, Castro LM, Hirata IY, et al (2010) CCP1/Nna1 functions in protein turnover in mouse brain: Implications for cell death in Purkinje cell degeneration mice. FASEB J 24: 1813–1823. 20061535 10.1096/fj.09-147942 PMC2874472

[19] Fukushima N, Furuta D, Hidaka Y, Moriyama R, Tsujiuchi T (2009) Posttranslational modifications of tubulin in the nervous system. J Neurochem. 109: 683–693. 10.1111/j.1471-4159.2009.06013.x 19250341

[20] Ikegami K, Setou M (2010) Unique post-translational modifications in specialized microtubule architecture. Cell Struct Funct 35: 15–22. 20190462 10.1247/csf.09027

[21] Janke C, Kneussel M (2010) Tubulin post-translational modifications: encoding functions on the neuronal microtubule cytoskeleton. Trends Neurosci 33: 362–372. 20541813 10.1016/j.tins.2010.05.001

[22] Rogowski K, van Dijk J, Magiera MM, Bosc C, Deloulme JC, et al (2010) A family of protein-deglutamylating enzymes associated with neurodegeneration. Cell 143: 564–578. 21074048 10.1016/j.cell.2010.10.014

[23] Berezniuk I, Vu HT, Lyons PJ, Sironi JJ, Xiao H, et al (2012) Cytosolic Carboxypeptidase 1 Is Involved in Processing alpha- and beta-Tubulin. J Biol Chem 287: 6503–6517. 22170066 10.1074/jbc.M111.309138 PMC3307270

[24] Campbell PK, Waymire KG, Heier RL, Sharer C, Day DE, et al (2002) Mutation of a novel gene results in abnormal development of spermatid flagella, loss of intermale aggression and reduced body fat in mice. Genetics 162: 307–320. 12242242 10.1093/genetics/162.1.307 PMC1462267

[25] Ikegami K, Heier RL, Taruishi M, Takagi H, Mukai M, et al (2007) Loss of alpha-tubulin polyglutamylation in ROSA22 mice is associated with abnormal targeting of KIF1A and modulated synaptic function. Proc Natl Acad Sci USA 104: 3213–3218. 17360631 10.1073/pnas.0611547104 PMC1802010

[26] Janke C, Rogowski K, van Dijk J (2008) Polyglutamylation: a fine-regulator of protein function? ‘Protein Modifications: beyond the usual suspects’ review series. EMBO Rep 9: 636–641. 18566597 10.1038/embor.2008.114 PMC2475320

[27] Louvi A, Grove EA (2011) Cilia in the CNS: the quiet organelle claims center stage. Neuron 69: 1046–1060. 21435552 10.1016/j.neuron.2011.03.002 PMC3070490

[28] Berbari NF, Kin NW, Sharma N, Michaud EJ, Kesterson RA, et al (2011) Mutations in Traf3ip1 reveal defects in ciliogenesis, embryonic development, and altered cell size regulation. Dev Biol 360: 66–76. 21945076 10.1016/j.ydbio.2011.09.001 PMC4059607

[29] Sharma N, Kosan ZA, Stallworth JE, Berbari NF, Yoder BK (2011) Soluble levels of cytosolic tubulin regulate ciliary length control. Mol Biol Cell 22: 806–816. 21270438 10.1091/mbc.E10-03-0269 PMC3057705

[30] Gerdes JM, Liu Y, Zaghloul NA, Leitch CC, Lawson SS, et al (2007) Disruption of the basal body compromises proteasomal function and perturbs intracellular Wnt response. Nat Genet 39: 1350–1360. 17906624 10.1038/ng.2007.12

[31] Haycraft CJ, Zhang Q, Song B, Jackson WS, Detloff PJ, et al (2007) Intraflagellar transport is essential for endochondral bone formation. Development 134: 307–316. 17166921 10.1242/dev.02732

[32] Chizhikov VV, Davenport J, Zhang Q, Shih EK, Cabello OA, et al (2007) Cilia proteins control cerebellar morphogenesis by promoting expansion of the granule progenitor pool. J Neurosci 27: 9780–9789. 17804638 10.1523/JNEUROSCI.5586-06.2007 PMC6672978

[33] Davenport JR, Watts AJ, Roper VC, Croyle MJ, van Groen T, et al (2007) Disruption of intraflagellar transport in adult mice leads to obesity and slow-onset cystic kidney disease. Curr Biol 17: 1586–1594. 17825558 10.1016/j.cub.2007.08.034 PMC2084209

[34] Che FY, Lim J, Pan H, Biswas R, Fricker LD (2005) Quantitative neuropeptidomics of microwave-irradiated mouse brain and pituitary. Mol Cell Proteomics 4: 1391–1405. 15970582 10.1074/mcp.T500010-MCP200

[35] Morano C, Zhang X, Fricker LD (2008) Multiple isotopic labels for quantitative mass spectrometry. Anal Chem 80: 9298–9309. 19551992 10.1021/ac801654h PMC2771887

[36] Berezniuk I, Sironi J, Callaway MB, Castro LM, Hirata IY, et al (2010) CCP1/Nna1 functions in protein turnover in mouse brain: Implications for cell death in Purkinje cell degeneration mice. FASEB J 24: 1813–1823. 20061535 10.1096/fj.09-147942 PMC2874472

[37] Pan H, Che FY, Peng B, Steiner DF, Pintar JE, et al (2006) The role of prohormone convertase-2 in hypothalamic neuropeptide processing: a quantitative neuropeptidomic study. J Neurochem 98: 1763–1777. 16903874 10.1111/j.1471-4159.2006.04067.x

[38] Zhang X, Che FY, Berezniuk I, Sonmez K, Toll L, et al (2008) Peptidomics of Cpe(fat/fat) mouse brain regions: implications for neuropeptide processing. J Neurochem 107: 1596–1613. 19014391 10.1111/j.1471-4159.2008.05722.x PMC2663970

[39] Wardman JH, Zhang X, Gagnon S, Castro LM, Zhu X, et al (2010) Analysis of peptides in prohormone convertase 1/3 null mouse brain using quantitative peptidomics. J Neurochem 114: 215–225. 20412386 10.1111/j.1471-4159.2010.06760.x PMC2897930

[40] Pazour GJ, Dickert BL, Vucica Y, Seeley ES, Rosenbaum JL, et al (2000) Chlamydomonas IFT88 and its mouse homologue, polycystic kidney disease gene tg737, are required for assembly of cilia and flagella. J Cell Biol 151: 709–718. 11062270 10.1083/jcb.151.3.709 PMC2185580

[41] Haycraft CJ, Swoboda P, Taulman PD, Thomas JH, Yoder BK (2001) The C. elegans homolog of the murine cystic kidney disease gene Tg737 functions in a ciliogenic pathway and is disrupted in osm-5 mutant worms. Development 128: 1493–1505. 11290289 10.1242/dev.128.9.1493

[42] Taulman PD, Haycraft CJ, Balkovetz DF, Yoder BK (2001) Polaris, a protein involved in left-right axis patterning, localizes to basal bodies and cilia. Mol Biol Cell 12: 589–599. 11251073 10.1091/mbc.12.3.589 PMC30966

[43] Seidah NG (2011) The proprotein convertases, 20 years later. Methods Mol Biol 768: 23–57. 21805237 10.1007/978-1-61779-204-5_3

[44] Mzhavia N, Pan H, Che FY, Fricker LD, Devi LA (2003) Characterization of endothelin-converting enzyme-2. Implication for a role in the nonclassical processing of regulatory peptides. JBiolChem 278: 14704–14711. 10.1074/jbc.M211242200 PMC3862352 12560336

[45] Wardman JH, Zhang X, Gagnon S, Castro LM, Zhu X, et al (2010) Analysis of peptides in prohormone convertase 1/3 null mouse brain using quantitative peptidomics. JNeurochem 114: 215–225. 20412386 10.1111/j.1471-4159.2010.06760.x PMC2897930

[46] Naggert JK, Fricker LD, Varlamov O, Nishina PM, Rouille Y, et al (1995) Hyperproinsulinemia in obese fat/fat mice associated with a point mutation in the carboxypeptidase E gene and reduced carboxypeptidase E activity in the pancreatic islets. Nature Genetics 10: 135–142. 7663508 10.1038/ng0695-135

[47] Zhang X, Che FY, Berezniuk I, Sonmez K, Toll L, et al (2008) Peptidomics of Cpe(fat/fat) mouse brain regions: implications for neuropeptide processing. JNeurochem 107: 1596–1613. 19014391 10.1111/j.1471-4159.2008.05722.x PMC2663970

[48] Reznik SE, Fricker LD (2001) Carboxypeptidases from A to z: implications in embryonic development and Wnt binding. Cell Mol Life Sci 58: 1790–1804. 11766880 10.1007/PL00000819 PMC11337317

[49] Arolas JL, Vendrell J, Aviles FX, Fricker LD (2007) Metallocarboxypeptidases: emerging drug targets in biomedicine. Curr Pharm Des 13: 349–366. 17311554 10.2174/138161207780162980

[50] Lyons PJ, Fricker LD (2010) Substrate specificity of human carboxypeptidase A6. J Biol Chem 285: 38234–38242. 20855895 10.1074/jbc.M110.158626 PMC2992257

[51] Tanco S, Zhang X, Morano C, Aviles FX, Lorenzo J, et al (2010) Characterization of the substrate specificity of human carboxypeptidase A4 and implications for a role in extracellular peptide processing. J Biol Chem 285: 18385–18396. 20385563 10.1074/jbc.M109.060350 PMC2881764

[52] Lyons PJ, Fricker LD (2011) Carboxypeptidase O is a glycosylphosphatidylinositol-anchored intestinal peptidase with acidic amino acid specificity. J Biol Chem 286: 39023–39032. 21921028 10.1074/jbc.M111.265819 PMC3234727

[53] Paturle-Lafanechere L, Manier M, Trigault N, Pirollet F, Mazarguil H, et al.. (1994) Accumulation of delta 2-tubulin, a major tubulin variant that cannot be tyrosinated, in neuronal tissues and in stable microtubule assemblies. J Cell Sci 107 1529–1543. 10.1242/jcs.107.6.1529 7962195

[54] Berbari NF, Sharma N, Malarkey EB, Pieczynski JN, Boddu R, et al (2013) Microtubule modifications and stability are altered by cilia perturbation and in cystic kidney disease. Cytoskeleton 70: 24–31. 23124988 10.1002/cm.21088 PMC3552319

[55] Ikegami K, Sato S, Nakamura K, Ostrowski LE, Setou M (2010) Tubulin polyglutamylation is essential for airway ciliary function through the regulation of beating asymmetry. Proc Natl Acad Sci USA 107: 10490–10495. 20498047 10.1073/pnas.1002128107 PMC2890849

[56] Vogel P, Hansen G, Fontenot G, Read R (2010) Tubulin tyrosine ligase-like 1 deficiency results in chronic rhinosinusitis and abnormal development of spermatid flagella in mice. Vet Pathol 47: 703–712. 20442420 10.1177/0300985810363485

[57] Wloga D, Dave D, Meagley J, Rogowski K, Jerka-Dziadosz M, et al (2010) Hyperglutamylation of tubulin can either stabilize or destabilize microtubules in the same cell. Eukaryot Cell 9: 184–193. 19700636 10.1128/EC.00176-09 PMC2805296

[58] Kim J, Lee JE, Heynen-Genel S, Suyama E, Ono K, et al (2010) Functional genomic screen for modulators of ciliogenesis and cilium length. Nature 464: 1048–1051. 20393563 10.1038/nature08895 PMC2929961

[59] O’Hagan R, Piasecki BP, Silva M, Phirke P, Nguyen KC, et al (2011) The tubulin deglutamylase CCPP-1 regulates the function and stability of sensory cilia in C. elegans. Curr Biol 21: 1685–1694. 21982591 10.1016/j.cub.2011.08.049 PMC4680987

[60] Rodriguez de la Vega Otazo M, Lorenzo J, Tort O, Aviles FX, Bautista JM (2012) Functional segregation and emerging role of cilia-related cytosolic carboxypeptidases (CCPs). FASEB J 27: 424–431. 23085998 10.1096/fj.12-209080

[61] Gaertig J, Wloga D (2008) Ciliary tubulin and its post-translational modifications. Curr Top Dev Biol 85: 83–113. 19147003 10.1016/S0070-2153(08)00804-1

[62] Fricker LD, Gelman JS, Castro LM, Gozzo FC, Ferro ES (2012) Peptidomic analysis of HEK293T cells: effect of the proteasome inhibitor epoxomicin on intracellular peptides. J Proteome Res 11: 1981–1990. 22304392 10.1021/pr2012076 PMC3315381

[63] Gelman JS, Sironi J, Berezniuk I, Dasgupta S, Castro LM, et al (2013) Alterations of the intracellular peptidome in response to the proteasome inhibitor bortezomib. PLoS One 8: e53263. 23308178 10.1371/journal.pone.0053263 PMC3538785

[64] Fricker LD (2010) Analysis of mouse brain peptides using mass spectrometry-based peptidomics: implications for novel functions ranging from non-classical neuropeptides to microproteins. Mol Biosyst 6: 1355–1365. 20428524 10.1039/c003317k PMC2902593

[65] Gelman JS, Sironi J, Castro LM, Ferro ES, Fricker LD (2011) Peptidomic analysis of human cell lines. J Proteome Res 10: 1583–1592. 21204522 10.1021/pr100952f PMC3070057

