# Supplementary figures and images for: Quantitative Peptidomics of Purkinje Cell Degeneration Mice
Source: PLoS One. 2013 Apr 8;8(4):e60981. doi: 10.1371/journal.pone.0060981 (PMC3620535; doi:10.1371/journal.pone.0060981)

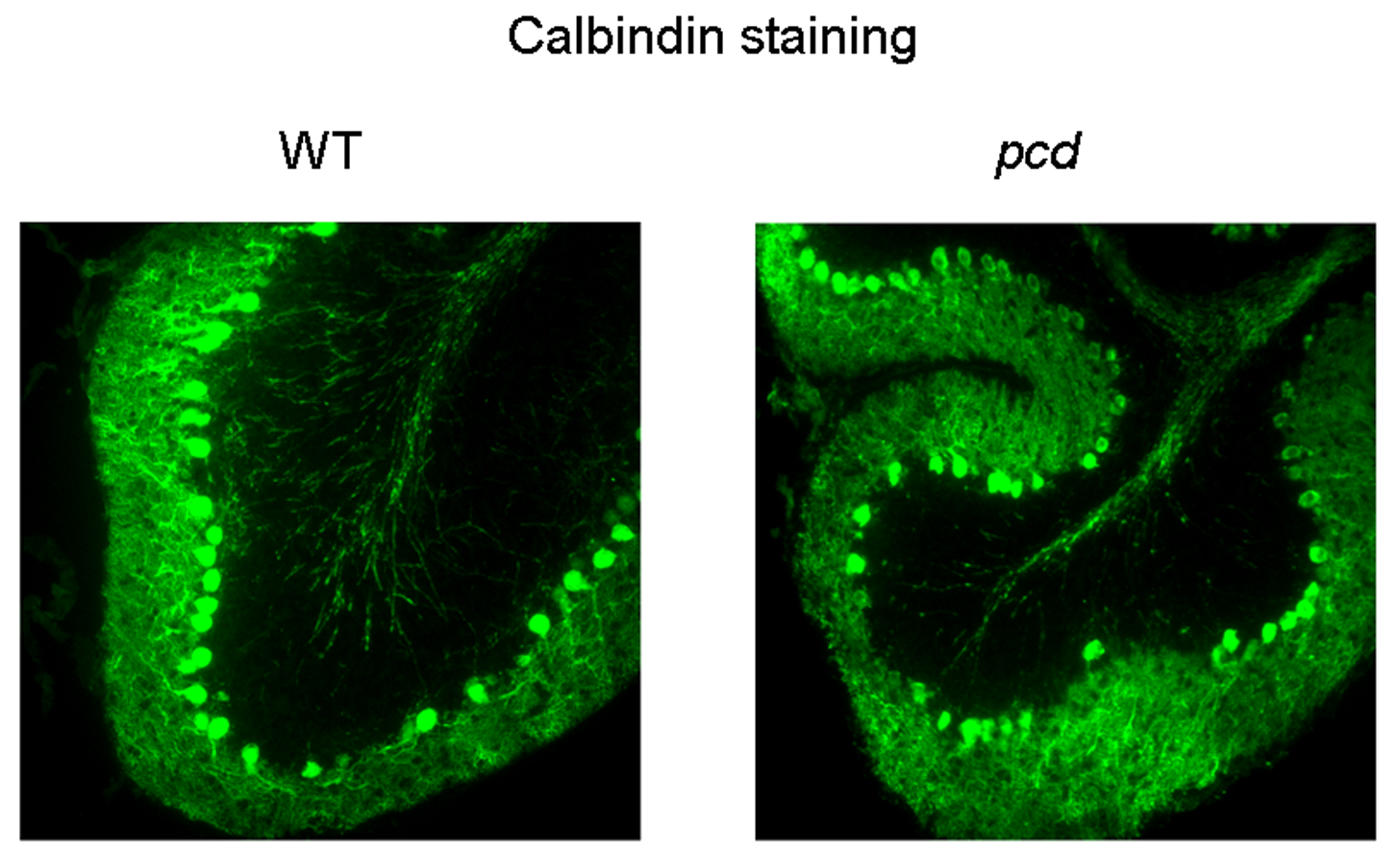

Supplement: Figure S1 — Distribution of calbindin staining in cerebellum of 3-week-old WT and pcd mice. WT and pcd cerebellar sections were probed with antibodies against calbindin, a marker for Purkinje cells. Staining shows Purkinje cell dendrites, Purkinje cell bodies, and Purkinje cell axons (all green) in WT (left panel) and pcd (right panel) cerebellum. (TIF) [file pone.0060981.s001.tif]
